# Exposure to *Phlebotomus argentipes* (Diptera, Psychodidae, Phlebotominae) Sand Flies in Rural Areas of Bihar, India: The Role of Housing Conditions

**DOI:** 10.1371/journal.pone.0106771

**Published:** 2014-09-03

**Authors:** Paritosh Malaviya, Epco Hasker, Albert Picado, Mukesh Mishra, Jean-Pierre Van Geertruyden, Murari Lal Das, Marleen Boelaert, Shyam Sundar

**Affiliations:** 1 Institute of Medical Sciences Banaras Hindu University, Varanasi, India; 2 Department of Public Health, Institute of Tropical Medicine, Antwerp, Belgium; 3 Centre de Recerca en Salut Internacional de Barcelona (CRESIB, Hospital Clínic-Universitat de Barcelona), Barcelona, Spain; 4 International Health Unit, University of Antwerp, Antwerp, Belgium; 5 B.P. Koirala Institute of Health Sciences, Dharan, Nepal; Centro de Pesquisas René Rachou, Brazil

## Abstract

**Background:**

Visceral Leishmaniasis (VL) is a vector-borne infectious disease, caused by the protozoan *Leishmania donovani*, which is transmitted by phlebotomine sand flies. In an earlier study in Bihar, India, we found an association between incidence of VL and housing conditions. In the current study we investigated the influence of housing structure and conditions in and around the house on the indoor abundance of *Phlebotomus argentipes*, the vector of VL in this area.

**Methods:**

In each of 50 study villages in Muzaffarpur district, we randomly selected 10 houses. Light traps were installed in each house for one night during three annual peaks of sand fly density over two successive years. Sand flies captured were morphologically identified and segregated by species, sex and feeding status. Data on housing conditions and socio-economic status were also collected. We fitted a linear mixed-effects regression model with log-transformed *P. argentipes* counts as outcome variable and village as random effect.

**Results:**

*P. argentipes* was found in all but four of the 500 households. There was considerable variability between the years and the seasons. On bivariate analysis, housing structure, dampness of the floor, keeping animals inside, presence of animal dung around the house, and socio-economic status were all significantly associated with sand fly density. Highest sand fly densities were observed in thatched houses. In the multivariate model only the housing structure and socio-economic status remained significant.

**Conclusions:**

Better housing conditions are associated with lower sand fly densities, independent of other socio-economic conditions. However, in this area in Bihar even in the better-built houses sand flies are present.

## Introduction

Visceral Leishmaniasis (VL) or kala-azar is an infectious disease caused by the protozoan parasite *Leishmania donovani*, which is transmitted by phlebotomine sand flies. The disease is fatal if left untreated [1] and has an estimated worldwide annual incidence of 200,000–400,000 cases, two thirds of which occur on the Indian subcontinent [2]. Bihar state of India accounts for more than 50% of the VL caseload in the subcontinent and for 90% of the VL caseload in India [3]. Three VL endemic countries - India, Nepal and Bangladesh- have committed to eliminate VL from the region by 2015. Their target is to reduce the annual VL incidence to less than one new case per 10,000 inhabitants in all endemic districts [4]. In India, the National Kala-azar Elimination Program has adopted two main strategies: early detection and treatment of cases in VL endemic districts and vector control using indoor residual spraying (IRS) of houses and cattle sheds. A better understanding of the determinants of indoor population density of *Phlebotomus argentipes*, the vector of VL in this region, is therefore important for the fight against VL.

Both male and female sand flies feed on plant sugars for energy and longevity, but the females require a blood-meal from a host animal in order to produce eggs. *Phlebotomus argentipes* may become infected with *L. donovani*, while feeding on an infected host (human) and may transmit the parasite when taking a subsequent blood-meal from a different host [5], [6]. It is assumed that humans are the only reservoir for *L. donovani* in the Indian subcontinent [1], though recently there have also been reports of infection in some domestic animals [7]. Sand flies are found resting mainly in cracks and crevices in the walls of houses and cattle sheds [8] and their density is influenced by meteorological and environmental conditions such as temperature and humidity [9], [10]. *Phlebotomus argentipes* density in Bihar shows a clear seasonality that is associated with outside temperature and rainfall [11]. In West Bengal, *P. argentipes* density was associated with environmental factors such as soil temperature and moisture [12].

VL is recognized as a disease of the poorest of the poor; VL affected communities are socially and economically deprived, which is patently reflected in their housing conditions [13], [27]. The influence of housing conditions and presence of cattle on the indoor sand fly population dynamics is complex and as yet not fully elucidated. *Phlebotomus argentipes* breed in moist organic soils at the junction of the floor and walls of cattle sheds and earthen houses, with a greater propensity to breed in cattle sheds than in human houses [14]. Mud-plastered walls with cracks, and damp floors in constructions close to small water bodies and vegetation, are assumed to be good breeding and resting sites [28]. A study in the Vaishali and Lohardagga district in Bihar found mud-plastered walls, mixed dwellings (animals and humans under the same roof) and geographic area associated with presence of the vector to be significant house-level risk factors associated with transmission of Indian kala-azar [15]. Another study in Bihar on the effect of house type on the sand fly density was not conclusive [5]. Housing conditions could also impact through another mechanism. In a study in Panama, where other phlebotomine sand fly species (*Lutzomyia panamensis, Lutzomyia triramula, Lutzomyia dysponeta, Lutzomyia trapidoi*, and *Lutzomyia gomezi*) are the vectors, destitute housing conditions were associated with reduced effectiveness of insecticide thermal fogging [16]. To further elucidate the influence of housing structure on sand fly density, we investigated the association between *P. argentipes* density and different types of housing as well as other conditions in and around the house, while controlling for potential confounding by socio-economic status.

## Materials and Methods

### Entomological monitoring

The study was conducted in 50 rural villages of Kanti and Marwan blocks of Muzaffarpur district in Bihar between July 2009 and April 2011. Ten households were randomly selected from each village. In each of these households one CDC light trap was set up for one night during each of the three seasons identified as peak sand fly density periods over two successive years; seasons selected were April/May (summer), July/August (rainy season) and October/November (start of winter) [5]. Thus, over periods of two years light traps were installed for six nights in each household. Light traps were installed in the main bedroom of the house and run from 6 pm in the evening to 6 am the next morning. Per night, light traps were installed simultaneously in 20 households in two villages. Consequently, for each season it took 25 nights to complete the process. The insects collected were morphologically identified [17], [18], [19] in the laboratory by a trained entomologist and grouped by species and subspecies (*P. argentipes*, *P. papatasi and P*. (*Sergentomyia*) sp.). Male and female sand flies were segregated and gonotrophic conditions of female sand flies were identified as blood fed, unfed or gravid. The total number of *P. argentipes* in each household was determined by aggregating the total numbers of males and females captured during the six nights of trapping over the two-year period.

### Household characteristics

During the second survey round in October/November 2009, we collected information regarding conditions of housing and of the immediate surroundings. This included information about main materials used in floor, walls and roof of the room in the house where the light trap was installed. We also recorded dampness of the floor, penetration of daylight, presence of windows and cross-ventilation. We checked for the presence of cooking stoves and whether animals were kept inside the house. For the analysis, houses were subdivided into four types based on structure of walls and floor. We distinguished between thatched houses, un-plastered brick houses and plastered brick houses; the latter category was sub-divided into houses with an earthen floor and houses with a cemented floor. A room was judged as cross-ventilated if there was at least one window in a wall other than the one in which the door was located. Dampness of floors was assessed by touching the floor with the back of the hand. We also collected information about water logging and about the presence of animal dung in the immediate surroundings of the house. Socio-economic status was assessed for the household based on a previously validated asset index [21], which included ownership of land, motorcycle(s), bicycle(s), television set(s), radio(s), mobile phone(s), watch (es), fan(s), mattress (es) and bed(s). Assets owned were converted into an assets score using principal-component analysis, the assets score was converted into an asset-index based on the five quintiles [13].

### Statistical analyses

The study data were entered into an MS Access database independently by two data entry clerks using a double-data entry system. All statistical analysis was performed in Stata/IC V10.1 (StataCorp., College Station, TX, USA). Taking into account potential clustering at village level, we fitted a linear mixed-effects regression model with ‘village’ as random effect [22]. To normalize the data and fulfill model assumptions we used a natural logarithm transformation of ln(y+1) [22]. A two-step procedure was used to select the most parsimonious model. First, all explanatory variables were assessed in a bivariate model with ‘village’ as random effect; variables significant at the p = 0.10 level were included in the final model. We then used a stepwise backward elimination procedure; variables with a p-value of greater than 0.05 were removed one at a time. We tested for statistically significant interactions among the variables retained in the final multivariate model; again a p-value of 0.05 was used as a threshold. To calculate the proportion of the variation in *P. argentipes* density explained by the variables retained as fixed effects in the final model, we determined the residual variance of the full model, and that of the null model with only the random effect (village). We then divided the difference between these two variances by the residual variance of the null model [29]. For the bivariate analysis we report coefficients with their p-values as well as median sand fly densities; for the final model we report the coefficients with their corresponding p-values and the mean *P. argentipes* densities predicted by the model for a village with average vector density.

To assess the association between presence of all three stages of female *P. argentipes* (fed, unfed and gravid) and factors retained in the final linear regression model, we recoded the presence of three stages into a binary variable (‘Yes’ if all three stages were present in the same household during the same trapping night, otherwise ‘No’) and fitted a logistic regression model with village as random effect.

### Ethical issues

We obtained ethical clearance for the study from the review committee of the U.S. National Institutes of Health (NIH), as well as the Institutional Review Board of the Institute of Medical Sciences, Banaras Hindu University, Varanasi, India. The IRB at Banaras Hindu University is registered with the US National Institutes of Health. The study and its procedures were explained to the heads of households and their prior informed written consent was obtained on a consent form. The procedure was approved by the Ethics Committee of Banaras Hindu University.

## Results

**Figure 1:**
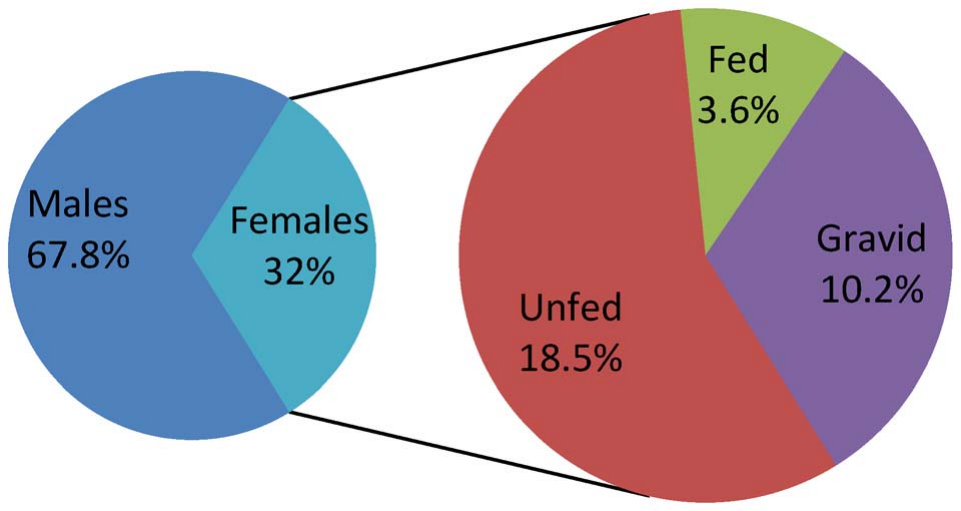
Cumulative distribution of *Phlebotomus argentipes* by sex and by feeding status among females.

**Figure 2:**
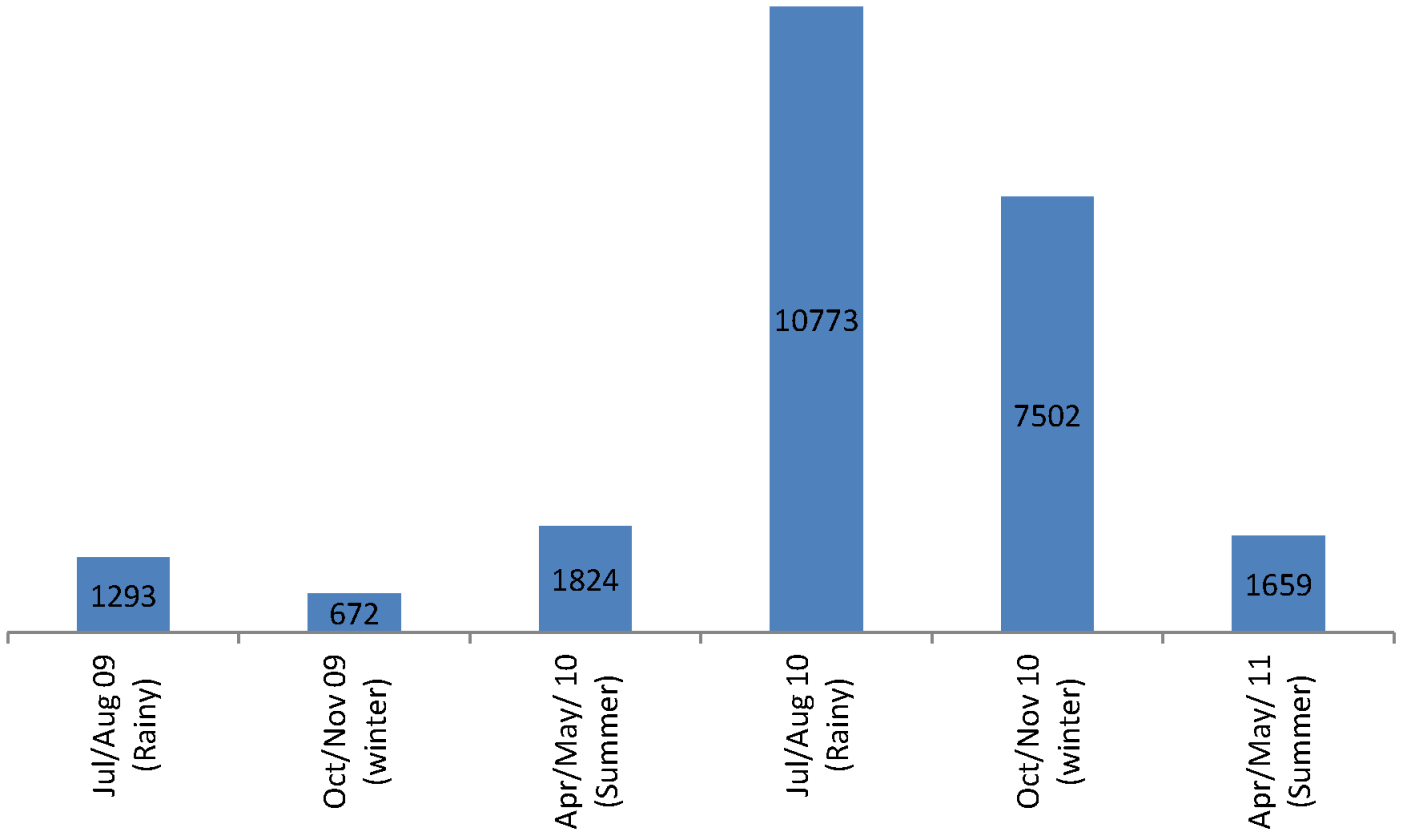
*Phlebotomus argentipes* abundance during three annual peaks of the two study years.

All the 500 households selected were sampled on six occasions as planned. We collected 49,126 sand flies of which 23,723 (48.3%) were *P. argentipes* (Table 1). *Phlebotomus argentipes* were captured from 496 of 500 houses; the median total yield per house (for six nights of sampling) was 25 *P. argentipes*, with a maximum of 681. Of the *P. argentipes* captured, 67.8% were males. Among females captured, 57.3% were unfed, 11.2% were fed, and 31.6% were gravid (Figure 1). *Phlebotomus argentipes* density strongly fluctuated from year to year and season to season. Highest densities were observed during rainy seasons (July-August) but there were significant annual fluctuations (Table 1). Major fluctuations in feeding status of female *P. argentipes* were observed in the rainy and winter seasons; for summers similar proportions were found across the years (Figure 2).

**Table 1:** Overview of *Phlebotomus argentipes* captured by sex and sampling period.

Survey	Males	Females			Total
		Unfed	Fed	Gravid	
Jul/Aug 09	884	306	81	22	1293
Oct/Nov 09	478	131	61	2	672
Apr/May/10	703	519	156	446	1824
Jul/Aug 10	7213	2275	357	928	10773
Oct/Nov 10	5706	928	120	748	7502
Apr/May/11	1089	221	78	271	1659
**Grand total**	**16073**	**4380**	**853**	**2417**	**23723**

One third of the study houses were thatched and *P. argentipes* density was highest in this type of house. Though we captured *P. argentipes* in almost all houses, density was higher in the households belonging to poorer wealth quintiles (Table 2). Other factors associated with *P. argentipes* density on bivariate analysis were dampness of the floor (p = 0.004), keeping animals inside (p = 0.002) and presence of animal dung around the house (p = 0.054). Each of these factors was associated with an increase in *P. argentipes* density. Significantly lower *P. argentipes* densities were observed in houses with windows (p<0.0005) and in houses with adequate ventilation (p<0.0005) (Table 3). When we repeated the analysis based on the total numbers of female *P. argentipes* sand flies only, results were similar (data not shown).

**Table 2:** Factors associated with *Phlebotomus argentipes* indoor density.

Factor		Number of households	Median (IQR) of total numbers of *P. argentipes* captured per household
Socio economic status*			
Group 1 (poorest)		101	35 (15–76)
Group 2		70	37.5 (19–68)
Group 3		98	24 (14–42)
Group 4		96	25 (11–57)
Group 5 (wealthiest)		117	17 (8–33)
Type of housing			
Thatched		161	41 (21–78)
Brick, un-plastered		150	22 (11–49)
Brick, plastered	Earth floor	113	18 (8–36)
	Cemented floor	76	17 (9–32.5)
Conditions in and around the house			
Traditional stove	Present	19	33 (17–76)
	Absent	481	24 (11–52)
Animals kept inside	Yes	20	24 (11–51)
	No	480	67 (30–107.4)
Penetration of sunlight inside	Yes	171	23 (12–53)
	No	329	25 (11–52)
Presence of windows	Yes	302	18 (10–38)
	No	198	40 (20–77)
Ventilation of room	Yes	193	18 (10–43)
	No	307	28 (14–63)
Damp floor	Yes	421	26 (12–55)
	No	79	17 (9–33)
Animal dung near house	Yes	485	14 (8–18)
	No	15	25 (12–53)

*n = 485.

**Table 3:** Factors associated with *Phlebotomus argentipes* indoor density, results of linear mixed effect regression models with village as random effect.

	Bivariate		Multivariate	
Factor	coefficient	p-value	coefficient	p-value
Socio economic status^1^				
Group 1 (poorest)	ref.		ref.	
Group 2	0.051	0.736	0.146	0.319
Group 3	−0.288	0.037	−0.187	0.162
Group 4	−0.282	0.046	−0.121	0.385
Group 5 (wealthiest)	−0.694	<0.0005	−0.379	0.010
Intercept^2^	3.554			
Type of housing				
Thatched	ref.		ref.	
Brick, un-plastered	−0.639	<0.0005	−0.592	<0.0005
Brick, plastered				
Earth floor	−0.845	<0.0005	−0.657	<0.0005
Cemented floor	−0.823	<0.0005	−0.605	<0.0005
Intercept^2^	3.797		3.831	
Conditions in and around the house				
Traditional stove present	0.169	0.48		
Intercept^2^	3.283			
Animals kept inside	0.716	0.002		
Intercept^2^	3.261			
Penetration of sunlight inside	−0.046	0.65		
Intercept^2^	3.305			
Presence of windows	−0.677	<0.0005		
Intercept^2^	3.698			
Ventilation of room	−0.440	<0.0005		
Intercept^2^	3.460			
Damp floor	0.363	0.004		
Intercept^2^	2.984			
Animal dung near house	0.542	0.054		
Intercept^2^	2.764			

1 n = 485.

2 mean (natural) log sand fly density of reference category.

Socio-economic status (p = 0.01 for poorest versus wealthiest quintile) and type of house (p<0.001 for thatched houses versus all other types) remained statistically significantly associated with *P. argentipes* density in multivariate analysis (Table 3). There was no interaction between the two factors (p = 0.81). Total counts of *P. argentipes* sand flies by socio economic group and type of housing as predicted by the multivariate model for a village with average vector density are shown in Table 4. Only 14% of the observed variability in *P. argentipes* density between households can be explained by the factors retained in the final model, i.e. housing conditions and socio-economic status.

**Table 4:** Total *Phlebotomus argentipes* counts by socio economic group and type of housing predicted by the multivariate model for a village with average vector density.

Housing type			Plastered brick	
Socio economic group	Thatched	Unplastered brick	Earth floor	Cemented floor
**Group 1 (poorest)**	46	26	24	25
**Group 2**	53	30	28	29
**Group 3**	44	21	20	21
**Group 4**	39	23	21	22
**Group 5 (wealthiest)**	27	17	16	17

The sub analysis examining whether or not all three feeding conditions of female *P. argentipes* (fed, unfed and gravid) were present in the household in at least one of six survey rounds showed that this was the case in 151 of 500 households. Presence of all three conditions was statistically significantly associated with type of housing. Controlling for socio-economic status, the odds of finding all three conditions in the same house during the same trapping night were more than three times higher in thatched houses than in plastered brick houses with cemented floors (Table 5).

**Table 5:** Odds of presence of three stages of female *Phlebotomus argentipes* in the same household at the same time as a function of socio economic status and type of housing (multivariate model with village as random effect).

Factor		OR (95% CI)
Socio economic status		
Group 1 (poorest)		ref.
Group 2		0.9 (0.4–1.8)
Group 3		0.9 (0.5–1.8)
Group 4		1.1 (0.6–2.2)
Group 5 (wealthiest)		0.5 (0.2–1.1)
Type of housing		
Thatched		ref.
Brick, un-plastered		0.5 (0.3–0.8)
Brick, plastered	Earth floor	0.4 (0.2–0.8)
	Cemented floor	0.3 (0.1–0.7)

## Discussion

In this study we showed that housing conditions are associated with sand fly density in Bihar, India, in particular thatched houses have higher numbers of *P. argentipes*. Independent of housing conditions, socio-economic status was also associated with vector density; the poorer the household, the higher its exposure to sand flies. Similar associations were observed for other phlebotomine sand fly species in Panama [16]. In an earlier study in the same area in Bihar we were able to show statistically significant associations between housing conditions and socio economic status on one side and VL on the other [21]. In that study we also showed that the coverage of indoor residual insecticide spraying was grossly inadequate and the current data seem to corroborate that. We were able to capture *P. argentipes* in all but four of the 500 study houses, including the more wealthy households.

Whereas differences in housing conditions are likely to reflect differences in exposure to *P. argentipes* sand flies, differences in socio-economic status may also reflect differences in nutritional status of the people and therefore propensity to progress to disease once infected [23]. *Phlebotomus argentipes* sand flies are predominantly peridomestic [24], [25] and are assumed to breed in the moist organic soil of cattle sheds and earthen houses, though the exact breeding sites of the sand flies need to be better documented [14]. Finding all three conditions of female *P. argentipes* (fed, unfed and gravid) in the same household indicates that these houses are likely to be diurnal resting or breeding sites [20]. In our study the odds were highest for thatched houses.

Only 14% of the total variability in indoor sand fly density is explained by housing conditions and socio-economic status, the two factors retained in the final model. Given the structure of the villages in this region where animals and organic waste are ubiquitous and all different varieties of houses can be found within a few meters of one another, it is not surprising that *P. argentipes* density does not differ much between households. There may also be other - extra-domiciliary -factors at play. These factors were not measured in our study. In a recent study in Bihar, sand flies were found to be most abundant in outdoor locations [5]. A cluster randomized trial of bed nets in India and Nepal also pointed to possible outdoor exposure to sand flies as an explanation for ongoing transmission [26].

## Conclusion

Better housing and higher wealth status are both independently associated with reduced indoor vector density. Efforts of the Indian government to improve housing conditions in VL endemic areas are commendable. However, as vectors are present even in the better houses; improving housing conditions will not necessarily preclude the need for additional measures such as indoor residual spraying or environmental sanitation.
